# Social entrepreneurial intention among university students in China

**DOI:** 10.1038/s41598-024-58060-4

**Published:** 2024-03-28

**Authors:** Xinyue Lyu, Abdullah Al Mamun, Qing Yang, Norzalita Abd Aziz

**Affiliations:** UKM - Graduate School of Business, Universiti Kebangsaan Malaysia (UKM), 43600 Bangi, Selangor Darul Ehsan Malaysia

**Keywords:** Entrepreneurial attitude, Values on sustainability, Opportunity recognition competency, Social entrepreneurial intention, University students, Human behaviour, Psychology and behaviour, Sustainability

## Abstract

The issues of employment, social inequality, and resource scarcity can be effectively addressed through social entrepreneurship, contributing to the growing research interest on the formation of social entrepreneurial intention. Using the theory of planned behaviour, the current study examined the influence of selected key factors on social entrepreneurial intention among university students. Based on the cross-sectional quantitative research design, online survey was conducted, which involved 684 students and graduates from five universities in Southern China. The obtained results demonstrated the significant and positive influence of perceived values on sustainability, opportunity recognition competency, attitude towards entrepreneurship, subjective norms, and perceived behavioural control on social entrepreneurial intention. Additionally, attitude towards entrepreneurship partially mediated the relationships of risk-taking propensity, self-efficacy, and need for achievement on social entrepreneurial intention. The multi-group analysis results also showed that need for achievement and social entrepreneurial intention differed significantly between genders, which provides new ideas for future investigations into the impact of gender on social entrepreneurial intentions. Based on the findings of this study, it is crucial that university students are exposed to relevant courses or training to develop social entrepreneurship competencies and promote sustainable values. The findings of this study will provide policymakers with relevant policy guidelines and more effective theoretical support to achieve the goal of promoting social entrepreneurship among university students in a more resource-efficient and effective manner.

## Introduction

With the rapid economic growth and technological advancements, various issues related to the environment, social inequality, resource scarcity, and high unemployment rate have become increasingly prominent, which pose critical concern for governments, profit and non-profit organisations, and various stakeholders. About two-thirds of countries and regions, including China, encounter social inequality, which would eventually lead to social inefficiency^1^. Through a market-based approach, social entrepreneurship creates both economic and social values to deal with numerous global social and environmental issues^2^.

Social entrepreneurship refers to the process of addressing environmental or social issues through market practices, and collaboration often takes place in communities with limited opportunities, resources, and rights^3^. Social entrepreneurs simultaneously pursue their economic and social goals and maintain the balance between both goals within their enterprise^4^. Unlike traditional entrepreneurship that focuses on maximising profitability, social entrepreneurship focuses on making the world a better place by reducing social problems^5^. Social entrepreneurship enhances the quality of life for underprivileged communities, establishes social inclusion, and promotes the engagement of products, services, and processes^6^. Social entrepreneurs contribute by acting as a support system, sharing knowledge, skills and experience in social enterprises and fulfilling their social mission by meeting the need to improve the quality of life in society^5^. Therefore, social entrepreneurs play critical roles of transforming the lives of underprivileged communities and dealing with issues of social inequality and other issues in society^7^. Accordingly, social entrepreneurship has continued to gain the attention of policymakers, practitioners, and researchers^8,9^.

University students and graduates are a potential emerging force in the social entrepreneurship market that cannot be ignored^10^. Studies on social entrepreneurship education in universities and its influence on students’ social entrepreneurship behaviour have gained growing popularity among researchers from the viewpoint of entrepreneurship education^11–13^. This may be attributed to the wide-ranging programmes offered at universities that influence students’ learning and career growth as an entrepreneur ^14^. Besides that, social entrepreneurship education can shape students’ sustainable development perspectives and values, as well as social entrepreneurial skills and nurture their social entrepreneurial behaviour, including social responsibility^15,16^. However, there are limited and rather outdated findings on the theories of social entrepreneurship education within the Chinese context, which result in inadequate theoretical support for the development and enhancement of students’ social entrepreneurial intention and behaviour^17^.

The strongest predictor of social entrepreneurial behaviour is social entrepreneurial intention^18^. Finding out what influences social entrepreneurial intentions is a key task^19^. A great deal of research has been devoted to exploring the causes of entrepreneurial intention formation through the theory of planned behaviour (TPB)^20–22^. According to TPB, attitudes towards entrepreneurship, subjective norms and perceived behavioural control can shape entrepreneurial intentions^23,24^. Similarly, this theory has been introduced into entrepreneurship research in China^25,26^. However, few scholars have explored the social entrepreneurial intention model in depth^27^. Moreover, although entrepreneurship research using TPB is extensive, it does not fully account for the types of people who have social entrepreneurship. In other words, there is still a lack of research that identifies the unique factors that lead people to have social entrepreneurial intentions.

A highly prominent role of social entrepreneurs is to use their capabilities to contribute to environmental (both economic and social) sustainability^28^. First, this suggests that social entrepreneurs generally have pro-social motivations and an orientation towards sustainability^29–31^. Most people who develop social entrepreneurial intentions are influenced by their own altruism and values^9^. In turn, altruism is often associated with the values of sustainable consumption, and even sometimes because sustainable values lead to the emergence of an altruistic component^32,33^. This may represent a positive relationship to be explored between values on sustainability and social entrepreneurial intentions, however this relationship is still unconfirmed in the literature. Secondly, the competencies of social entrepreneurs are also a focus of interest. We have focused on the ability to identify opportunities, as opportunity identification is one of the most important barriers to entrepreneurship^34^. However, when exploring the formation of social entrepreneurial intentions, many scholars have focused on the competencies that come with experience or have generalized and explored all skills and abilities as entrepreneurial competencies^7,35^. Hence research on the impact of opportunity recognition competency on social entrepreneurial intention is instead missing. For these reasons, two variables, perceived values on sustainability and opportunity recognition competency, were introduced to complement the TPB’s use in studying social entrepreneurial intentions.

Based on a sample of university students and graduates in China, the current study which using PLS-SEM firstly examined factors that influence social entrepreneurial intention from TPB, namely attitude towards entrepreneurship, subjective norms, and perceived behavioural control, as well as the mediating effects of attitude towards entrepreneurship on the relationships of risk-taking propensity, self-efficacy, and need for achievement with social entrepreneurial intention. Secondly, the study added two influences—perceived values on sustainability and opportunity recognition competency—as a complement to the theory of planned behaviour. In addition, the study examined a gender comparative study in the hope of finding differences between males and females in the process of forming social entrepreneurial intentions. Findings on factors that influence social entrepreneurial intention may help decision-makers motivate university students to become social entrepreneurs now or in the future. The study was expected to present significant theoretical guidance and recommendations for policymakers, university educators, and social entrepreneurs to create a supportive environment for social entrepreneurship.

Overall, this paper is organised into several key sections. The subsequent section presents the review of related literature on the theory of planned behaviour (TPB) and its theoretical assumptions. Following that, hypotheses were proposed for testing. The obtained results and findings are discussed in the subsequent section. Conclusions and limitations of study are presented at the end of this paper.

## Literature review

### Social entrepreneurship in China

China’s nonprofit sector is heavily influenced by the state, which distinguishes it from most economies because of its deep political embeddedness^36^. The endeavor to transition to a market economy creates a unique environment for social entrepreneurs^37^. China has witnessed significant growth in social entrepreneurship over the past decade, recognizing it as an innovative approach to address economic and societal challenges in developing countries like China^38^.

Although there is currently no official certification channel for social enterprises in China, numerous certified social organizations exist^39^. These include rural cooperatives, rural enterprises, for-profit entrepreneurs participating in the Guangcai/Glorious Program, and nonprofit organizations, all of which exhibit characteristics akin to social enterprises^40^. However, the insufficiency of government procurement and subsidies as sustainable funding sources for many social organizations' daily operations has led to the adoption of simultaneous registration of commercial enterprises as a viable solution^39^. In this context, Farhoud et al.^41^ have also proposed that social enterprises should encompass entities within the non-profit sector, the for-profit sector with a social mission, and hybrid organizations.

According to the Global Entrepreneurship Monitor’s 2023–2024 Global Report (GEM 2024), over 60% of startups in China prioritize their societal and environmental impact, thus highlighting Chinese startups' significant concern for addressing social issues. However, despite the rapid development trend of social entrepreneurship, there are few studies specifically focused on social entrepreneurship within the Chinese context^42^. Most scholars concentrate on aspects such as the development of social entrepreneurship, economic benefits associated with social enterprises, and the role of entrepreneurial education in fostering social entrepreneurship^43–46^. A limited number of studies have also examined social entrepreneurial intention (SEI), such as Yang et al.^38^, who investigated the impact of values, beliefs, and norms on the social entrepreneurial intentions of employed individuals using the Values-Beliefs-Norms model. Choi et al.^47^, utilizing data from senior college students at a Sino-American university in China, explored changes in SEI across gender prior to and during the COVID-19 pandemic, as well as the influence of environmental and personal factors on SEI. However, there is significant potential for research to uncover the precursors of SEI. The study aims to explore Chinese university students’ and graduates’ perceived values on sustainability, opportunity recognition competency, attitude towards entrepreneurship, subjective norms, and perceived behavioural control on SEI based on TPB with an aim to enhance SEI research in China.

### Theoretical foundation

TPB has been widely used as the underlying theoretical basis for studies on entrepreneurial behaviour^48^. According to TPB, intention predicts the actual behaviour and elucidates the extent of efforts one makes to execute a planned action^49^. Ajzen^48^ suggests that in most cases, people’s behaviour is not entirely voluntary, but rather under control. Such influences from oneself, society and perception lead people to develop a particular plan aFnd then form the intention to carry it out before implementing the action. TPB is commonly used as a framework to measure intention and behaviour across various contexts^17^.

According to TPB, attitude, subjective norms, and perceived behavioural control shape one’s intention or in other words, one’s tendency to perform a specific behaviour^50^. Nasar et al.^49^ defined attitude as one’s way of thinking, feeling, or opinion about a specific behaviour or an activity related to personal preferences, strengths, weaknesses. Meanwhile, Gorgievski et al.^24^ defined subjective norms as one’s perceptions of the social support or pressure received when one engages in a certain behaviour or activity. Perceived behavioural control refers to the cognitive evaluation of one’s ability to execute a specific activity; in this case, this construct reflects one’s awareness of the skills required for entrepreneurship^51^.

TPB was considered the underlying theoretical basis for the study of entrepreneurial behaviour. According to TPB, university students' attitudes towards entrepreneurship (ATE), subjective norms (SUN), and perceived behavioural control (PBC) can positively influence their entrepreneurial intentions^52^. Individuals who show an interest in entrepreneurship usually listen to the advice of those around them, and receiving feedback may influence their decision to accept entrepreneurial activities based on attitudes, SUN, and PBC^22^. One of the benefits of TPB is that its original structure can be altered to suit more precise research domains and improve the quality of acceptable results, making the TPB model attractive for SEI research^53^. In particular, the study examined the influence of perceived value on sustainability (PVS), opportunity recognition competency (ORG), ATE, SUN, and PBC on SEI. The addition of the variables PVS and ORG, which complement the TPB in this study, allows for a more detailed and varied approach to improving SEI in terms of sustainable values and personal capabilities. Furthermore, ATE was conceptualised into risk-taking proclivity, self-efficacy, and the need for achievement to be discussed in greater depth from a psychological standpoint.

### Development of hypotheses

#### Risk-taking propensity (RTP) and attitude towards entrepreneurship (ATE)

Risk-taking propensity describes one’s willingness to take risks^54^. Entrepreneurs, especially of those without entrepreneurial experience or high education level, need to have significantly high risk tolerance^55^. Starting a new start-up with limited resources and unknown market potential comes with the risk of failure that may result in psychological risk and even health risk^56^. It has been found high risk-taking propensity among university student entrepreneurs^57^. University students who choose to be an entrepreneur, instead of securing a job with stable income, are already taking the risk^58^. Those who are not willing to take the risk due to the fear of failure are likely to overlook certain opportunities^59^. Risk-averse individuals are more likely to be resistant against entrepreneurship opportunities since they cannot afford the stress of failure, whereas individuals with risk-taking propensity tend to be more confident and better equipped to respond and make decisions to achieve entrepreneurial success^60^. Moreover, students' risk-taking motivation and behaviour were significantly and positively associated with their determination to undertake social entrepreneurship^61,62^. In view of the above, the following hypothesis was proposed for testing in the current study:

##### H1

RTP positively influences ATE.

#### Self-efficacy (SEF) and attitude towards entrepreneurship (ATE)

Bandura^63^ derived self-efficacy from the social cognitive theory and postulated its formation through mastery experience, social modelling, and social persuasion. According to Bandura^63^, self-efficacy determines the perceived self-enabling or self-diminishing manner and influences one’s decision-making. Meanwhile, Udayanan^64^ described self-efficacy as one’s belief that he or she can successfully perform a task. Self-efficacy can also explain one’s entrepreneurial attitude^65^. Individuals with self-efficacy are more likely to exhibit entrepreneurial potential^66^. Entrepreneurs with higher self-efficacy demonstrate higher confidence to succeed, whereas entrepreneurs with lower self-efficacy demonstrate lower confidence and take the next under the guidance of others^67^. Low entrepreneurial confidence potentially causes one to have poor commitment in entrepreneurship or even give up entirely. For example, as social issues are quite complex, if the self-efficacy of potential social entrepreneurs is too low, it may constitute a psychological barrier^68^. Increasing perceived self-efficacy serves as a crucial goal in entrepreneurship education for university students, enabling them to strengthen their beliefs in entrepreneurship and receive psychological and emotional support^69^. Individuals with higher perceived self-efficacy in entrepreneurship demonstrate more passion in entrepreneurship and positive attitude towards entrepreneurship^70^. Thus, the following hypothesis was proposed for testing in this study:

##### H2

SEF positively influences ATE.

#### Need for achievement (NFA) and attitude towards entrepreneurship (ATE)

According to the achievement theory, the strength of need for achievement affects one’s motivation and performance^71^. Yohana and Salsabila^72^ defined need for achievement as the motivation to achieve and be successful in a set of legal relationships. The existing literature on entrepreneurship has identified need for achievement as a determinant of entrepreneurial success^73^. Several prior studies highlighted need for achievement as the most crucial predictor of entrepreneurial intention^74^. Being a social entrepreneur is inherently about the pursuit of economic benefits and the achievement of addressing social inequalities^75^. The greater the need for achievement, the more eager entrepreneurs are to take risks^76^. Furthermore, students whose achievement needs are highly satisfied feel better psychologically and are able to consciously learn and exercise the competencies needed to start and develop new social enterprises^77^. All these facts suggest that higher achievement needs inspire more positive attitudes towards entrepreneurship^65,78,79^, whether for social entrepreneurs or for entrepreneurs in general. In view of the above, the following hypothesis was formulated:

##### H3

NFA positively influences ATE.

#### Perceived values on sustainability (PVS) and social entrepreneurial intention (SEI)

The concept of sustainability has recently gained significant attention, and its concept varies in different contexts. Jaramillo et al.^80^ described values on sustainability as the principle of simultaneously enhancing one’s competitive advantage and monetary or non-monetary benefits. From a business perspective, Faulkner and Badurdeen^81^ defined sustainability as making rational economic decisions in the selection of production systems that can reduce adverse implications on the environment and consider the conservation of energy and natural resources. Meanwhile, Husgafvel^82^ defined social sustainability as a standard social sustainable practice or performance related to the society, organisation, business, group, individual, product/service/process, or an activity (or activities). Thus, the current study viewed perceived values on sustainability as the principle related to sustainability practices with the emphasis on pro-environmental behaviour.

Values influence how individuals become what they favour or perceive and reflect how individuals view a certain subject or matter^83^. As consumers, perceived values effectively predict behavioural intention^84^. For instance, diners with perceived values on sustainability are generally more concerned about the environment and less likely to waste food^85^. When talking about social entrepreneurship, it is often associated with social responsibility and the creation of sustainable values^86^. Social entrepreneurs place substantial concern on sustainability^87^. They have a mindset of concern for social and environmental issues and are able to integrate sustainability values into their company's mission and work with other indicators related to entrepreneurial activities^88^. These studies suggest a potential positive correlation between sustainable values and SEI. Moreover, based on the perceived values on sustainability, individuals emphasise combining social, environmental, and economic values with the emphasis of creating social and environmental values, and these positive perceptions are positively related to the intention of starting a sustainable business^89^. In view of the above, although there was no evidence of the direct relationship between PVS and SEI, the following hypothesis was proposed for testing:

##### H4

PVS positively influences SEI.

#### Opportunity recognition competency (ORG) and social entrepreneurial intention (SEI)

As one of the key concepts in entrepreneurship, opportunity recognition is a process of discovering and understanding a certain change and deciding whether to act on the change^90^. Opportunity recognition competency is a specific ability that involves identifying and developing ideas, which is a criterion to the process of recognising an opportunity^91^. Recognising business opportunities has long been recognised as a criterion to realising entrepreneurship^92^. Several prior studies demonstrated the positive influence of opportunity recognition competency on students’ entrepreneurial intention^79,91^. However, the influence of opportunity recognition competency on social entrepreneurial intention has remained underexplored.

According to Trajano et al.^93^, the unmet social needs highlighted by community volunteers are identified as business opportunities, which then motivate the formation of social entrepreneurial intention. Social entrepreneurs also aim to meet unmet social needs by providing solutions and creating social value^31^. Basically, these volunteers and social entrepreneurs share similar attributes. Before they decide to meet social needs, they should first identify them^31^. It suggests that having the ability to identify social needs and turn them into useful business opportunities is necessary for those intending to engage in social entrepreneurship. Hoong et al.^94^ shared similar view on the significance of opportunity recognition competency as a determinant that can enhance one’s social entrepreneurial intention. It is also important to note that there are some differences between social entrepreneurs and general entrepreneurs in terms of identifying opportunities. Social entrepreneurs identify opportunities based on their observation of the social issues, while others may only consider these issues as threats to their business^95^. With that, the following hypothesis was proposed for testing:

##### H5

ORG positively influences SEI.

#### Attitude towards entrepreneurship (ATE) and social entrepreneurial intention (SEI)

According to TPB, attitude towards a specific behaviour describes one’s (favourable or unfavourable) evaluation of the results of the behaviour^48^. Attitude can also be viewed as the outcome of one’s evaluation of whether a certain behaviour aligns with personal beliefs. Attitude has also been regarded as a predictor of intention^50^. Tuan and Pham^68^ described attitude as an evaluation of an action, specifically on whether the outcomes of the action would influence (increase or reduce) the propensity of executing the action. In the case of entrepreneurship, Liñán and Chen^96^ described attitude towards entrepreneurship as personal evaluation of whether one can become an entrepreneur. Meanwhile, Anderson^97^ identified attitude towards entrepreneurship as an important determinant of entrepreneurial intention.

Studies have highlighted the translation of favourable evaluation of becoming an entrepreneur into entrepreneurial intention^78^. In the context of social entrepreneurship, attitudes towards entrepreneurship are an assessment of action^98^. In other words, an individual with positive attitude towards entrepreneurship is more likely to be involved in social entrepreneurship^99^. Similarly, students are highly likely to have a desire to become social entrepreneurs if they have a high level of desire to engage in social entrepreneurial behaviour^68^. When individuals with favourable attitude towards entrepreneurship receive support from the society, they are likely to possess the emotional strength to strengthen their social entrepreneurial intention^19^. With that, the current study formulated the following hypothesis for testing:

##### H6

ATE positively influences SEI.

#### Subjective norms (SUN) and social entrepreneurial intention (SEI)

As part of normative beliefs in TPB, the construct of subjective norms reflects the perceived social pressure and motivation to execute a certain behaviour based on the evaluation of the significant others^100^. Apart from establishing one’s perception of oneself, personal beliefs, and expected results, it can influence the one’s intention towards a particular behaviour^101^. When an individual receives the approval and support from the significant others (e.g., family, friends, or society) to perform a certain activity, the individual is likely to form the intention of performing the activity.

Social entrepreneurs rely heavily on social network connections to pursue their social mission, which leads to the possibility that their background and the people around them may play a significant role in their thoughts and behaviours^102^. Yang et al.^103^ found higher tendency of forming social entrepreneurial intention among potential social entrepreneurs within the collectivist cultural context in China than those in the United States. Tiwari et al.^99^ observed similar outcomes on the positive influence of subjective norms on social entrepreneurial intention among students within the collectivist context in India. In another study, Mamun et al.^104^ identified subjective norms as a significant determinant of students’ entrepreneurial intention. These studies presented notable findings that suggest the strong correlation between subjective norms and social entrepreneurial intention within the Chinese cultural context. Thus, the current study tested the following hypothesis based on a sample of Chinese students:

##### H7

SUN positively influences SEI.

#### Perceived behavioural control (PBC) and social entrepreneurial intention (SEI)

Ajzen^48^ described perceived behavioural control as the extent of perceived ease or difficulty in executing a certain behaviour. It describes one’s belief of whether one has the capacity to perform a specific activity^105^. Studies have used perceived behavioural control as a predictor of intention and actual behaviour^106–108^. With that, the current study viewed perceived behavioural control as the extent of one’s perceived ease or difficulty in starting a business and becoming an entrepreneur.

Studies on entrepreneurship have demonstrated the significance of perceived behavioural control. For instance, Joensuu-Salo et al.^109^ positive found strong correlation between entrepreneurial ability and perceived behavioural control and the influence of gender on the relationship between entrepreneurial ability and perceived behavioural control. Several prior studies showed the formation of perceived behavioural control prior to entrepreneurial intention^110,111^. On a similar note, Tiwari et al.^112^ highlighted perceived behavioural control as an essential factor that can positively influence social entrepreneurial intention. However, a recent study by Barba-Sánchez et al.^113^ suggests that the positive impact of PBC on entrepreneurial intentions is completely overshadowed by attitudes towards entrepreneurial behaviour, contrary to most views. Nevertheless, the study continues to argue that PBC can directly and positively influence SEI in a social entrepreneurship perspective. Therefore, the following hypothesis was proposed for testing in the current study:

##### H8

PBC positively influences SEI.

#### Mediating effects of ATE

The previous section discussed the positive influence of RTP, SEF, and NFA on ATE and the positive influence of ATE on SEI. Therefore, the current study postulated the potential mediating effects of ATE on the relationships of RTP, SEF, and NFA with SEI. RTP is one of the important dimensions in entrepreneurial activity due to the high level of uncertainty^57^. Any entrepreneur is prepared to take risks before deciding to start a business^55^. Although social entrepreneurs are more risk-averse than general entrepreneur^114^, Hossain^115^ still found that potential social entrepreneurs would have a higher propensity to take risks. Moreover, a higher risk-taking mentality positively fosters positive attitudes towards entrepreneurship and future perceptions of starting a social venture among university students^61^. Chipeta and Surujlal^116^ had found that both RTP and ATE can positively influence SEI after testing with a multiple linear regression equation. Tu et al.^61^ also showed that the greater the motivation of graduate students to take risks, the higher their ATE and SEI, and that the relationship between ATE and SEI was also positively significant. However, none of studies directly verified the mediating role of ATE.

Generally speaking, the stronger an entrepreneur's SEF is, the more entrepreneurial he/she will be and the more likely he/she will have the intention to start a business^117^. A high level of SEF creates a positive attitude towards entrepreneurship^65^. SEF helps an individual to generate social entrepreneurial intentions by showing him/her the feasibility of creating a social venture^118^. The stronger the SEF on social entrepreneurship, the more confident entrepreneurs are in recognising the opportunities for social change and the more likely they are to commercialise their social enterprise ideas^119^. In a more recent studies, the direct and significant influence of SEF on SEI has been verified^120,121^. In studies of entrepreneurship, ATE has been found to be an intermediate variable between SEF and entrepreneurial intentions^122^. It has also been suggested that individuals' behavioural attitudes and entrepreneurial attitudes can mediate SEF and SEI^68,105^. In conjunction with the aforementioned literature review, there is reason to believe that there is also a mediating role for ATE in SEF and SEI.

NFA is associated with the possibility of engaging in entrepreneurial activities, demonstrating an individual's ambition to achieve, acquire skills and achieve challenging goals^123^. Entrepreneurs always have a higher NFA than other professions and it is a driving force for their entrepreneurial intentions^124^. Enhancing students’ NFA can motivate students to choose entrepreneurship as a career^76^. NFA can also be described as a desire to do better and be more adventurous^125^. From these aspirations to becoming entrepreneurial ideas, a good entrepreneurial attitude is needed to maintain creativity, innovation and problem-solving skills^125^. Barton et al.^126^ state that motivated by the desire to achieve, students develop the will to form a social enterprise to fulfil the desire for self-fulfilment and autonomy. Combined with the above literature, NFA has the potential to motivate students to SEI by generating positive ATE.

Thus, the current study tested the following hypotheses:

##### H9a–c

ATE mediates the relationship between RTP, SEF and NFI on SEI.

**Figure 1:**
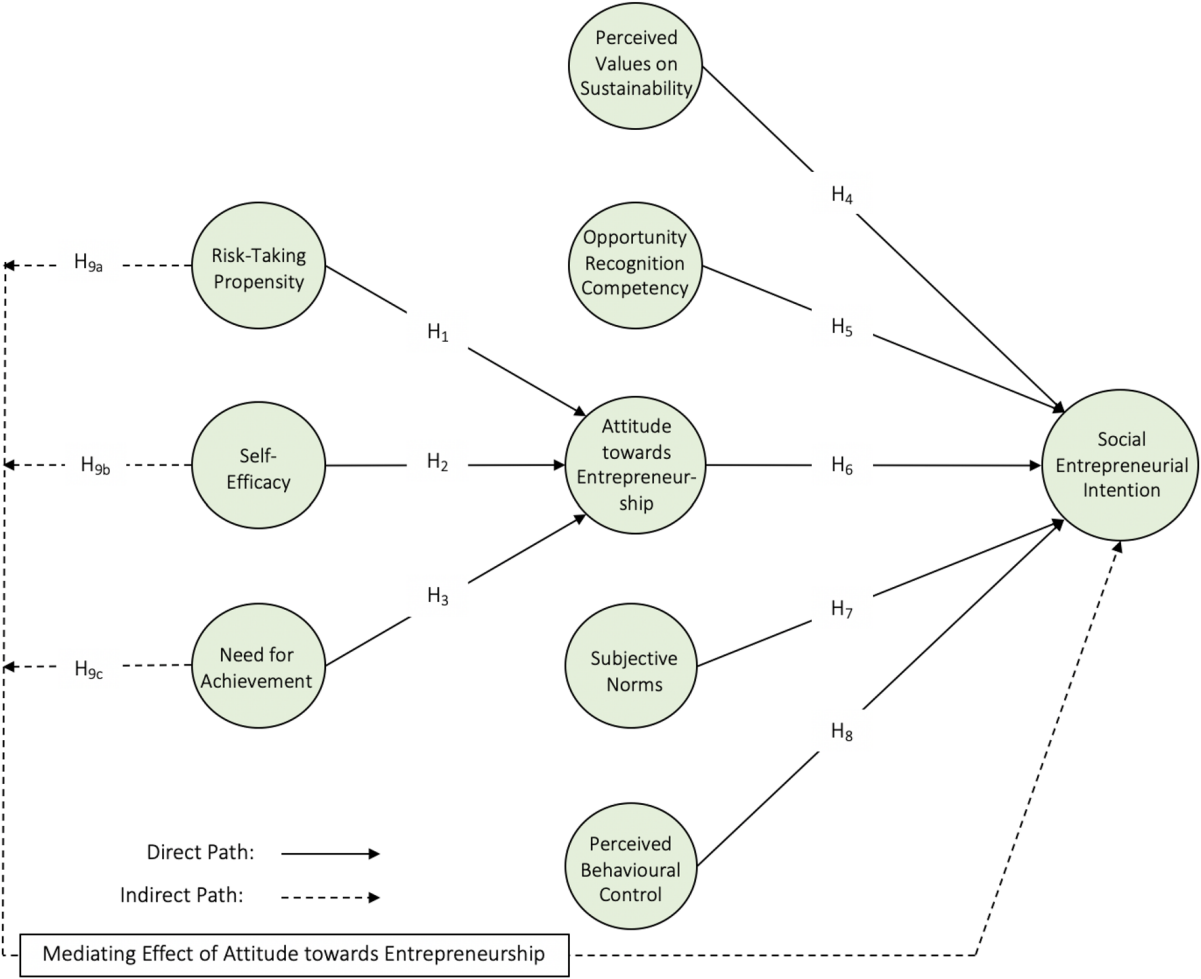
Research framework.

All association hypothesized above are presented in Fig. 1 below:

## Methodology

### Sample selection and data collection

The current study targeted current students or graduates of one private university and four public universities in Southern China. They include one university of Project 985, two Double First-Class universities but not in Project of 985, one First tier university and one Second tier university. The use of self-administered questionnaire survey and cross-sectional data was deemed adequate for the current study to examine factors that influence SEI. Moreover, the self-administered approach has been widely used to explore entrepreneurial intention^104,120^. We released this survey in June 2022 and concluded respondent recruitment and questionnaire collection in July 2022. First, we collected a sample larger than 100 in each university where possible, as Reinartz et al.^127^ proposed a minimum threshold of 100 samples for structural equation modelling via partial least squares (PLS-SEM). Respondents from each university were sampled via the convenience sampling techniques. The universities of these respondents were located in South China, but they were from almost all over the country.

G*Power (G*Power version 3.1.9.7) was used in this study to determine the minimum sample size required for the current study (effect size of 0.15, alpha of 0.05, power of 0.95 and 8 latent variables) and the results were calculated to indicate a minimum sample size of 160^128^. Also, referring to the suggestion of Gefen et al.^129^, the sample size should ideally be 10 times larger than the total number of items in the questionnaire. Based on the 48 items of the questionnaire involved in this study, the recommended sample size is at least 480 respondents. To ensure that a sufficient sample size was available for analysis after screening out non-useful responses, this study conducted a cross-sectional study to collect data in a specific population through an online questionnaire, resulting in 805 questionnaires. After data screening to remove responses that were under the age of 18 and incomplete responses to the informed consent form, only 684 questionnaires were used for this study.

### Instrument

The questionnaire comprised two main components. The first component gathered information on respondents’ demographics, including gender, age, education level, and household income. The second section discusses the items used to evaluate all variables in this study. RTP was measured using items provided by Mahmood et al.^78^, Mamun et al.^104^, and Wiramihardja et al.^79^. SEF items were adapted from Mahmood et al.^78^. Items for measuring NFA were taken from Mahmood et al.^78^ and Wiramihardja et al.^79^. The questions used to assess PVS were taken from Han et al.^130^ and Kim et al.^85^. ORG items were adopted from Lim et al.^91^ and Wiramihardja et al.^79^, whereas ATE items were adapted from Mahmood et al.^78^, Mamun et al.^104^, and Wiramihardja et al.^79^. Mahmood et al.^78^ and Mamun et al.^104^ provided the items used to assess SUN. Mamun et al.^104^ described the items used to assess PBC. Finally, we used SEI elements from Hockerts^131^ and Mahmood et al.^78^. The prior study validated these items, assuring the questionnaire's validity and removing the requirement for a pre-test in this investigation. Participants were asked to rate their comments on a five-point Likert scale, from ‘strongly disagree’ (1) to ‘strongly agree’ (5). The supplementary material includes all of the items utilized in this study (Table S1. Survey Instrument).

### Common method bias (CMB)

Common method bias refers to the perceived artificial covariation between the predictor and calibrator variables due to the same data source, measurement setting, as well as the context and characteristics of the items. Systematic error occurs under certain circumstances, such as the propensity of respondents to provide consistent or similar responses across measures due to the structure, wordings, or proximity of items and the similarity of time, medium, or place of data collection^132^. In order to minimise CMB, all items were meticulously constructed, and all respondents were properly briefed on the anonymity and confidentiality of their data and responses and the absence of specific (correct or incorrect) answers to the questions. Harman’s^133^ single-factor test was also conducted, which revealed negligible effect of CMB (34.259% < threshold of 50%). Table 1 presents the variance inflation factor (VIF) of each construct—all recorded values did not exceed 3.3^134^. In other words, there was no multi-collinearity issue.

**Table 1:** Full collinearity test.

Variables	RTP	SEF	NFA	PVS	ORG	ATE	SUN	PBC	SEI
VIF	1.610	1.522	1.562	1.300	1.443	1.700	1.783	1.717	1.782

*RTP* risk-taking propensity, *SEF* self-efficacy, *NFA* need for achievement, *PVS* perceived values on sustainability, *ORG* opportunity recognition competency, *ATE* attitude towards entrepreneurship, *SUN* subjective norms, *PBC* perceived behavioural control, *SEI* social entrepreneurial intention.

### Multivariate normality

The online Web Power tool (https://webpower.psychstat.org/models/kurtosis/) was used in this study to validate the multivariate normality of the acquired data despite the absence of such criterion for the analysis (PLS). Based on the obtained results, Mardia’s multivariate skewness and kurtosis *p*-values did not exceed 0.05, suggesting non-normality.

### Data analysis

Partial least squares structural equation modelling (PLS-SEM) has gained increasing use in social science research. Unlike covariance-based SEM (CB-SEM), PLS-SEM relies on the data variance and evaluates the model parameters based on total variance. PLS-SEM is recommended for both small and large data that are not normally distributed and the presence of complex structural models with multiple structures, indicators, and relationships^135^. Although PLS-SEM only provides standard linear models^135^, which may not be sufficient to face the complexity of social sciences, considering that the main purpose of the study is to predict and explain the target structure, PLS-SEM was performed.

### Ethical approval and consent to participate

The Human Research Ethics Committee of Guangxi Medical University approved this study (Ref. No. 2023–0227). This study has been performed in accordance with the Declaration of Helsinki. Written informed consent for participation was obtained from respondents who participated in the survey. No data was collected from anyone under 18 years old.

## Results

### Demographic characteristics

Referring to Table 2, the majority of the respondents were female respondents (55.0%). Besides that, 84.2% of the total respondents were undergraduate students. Most of these respondents were of the age group of between 23 and 26 (32.7%). About 41.4% of the total respondents reported attaining household income of between CNY 7501 and CNY 10,000. Additionally, 70.5% of the total respondents reported to be in the field of social science.

**Table 2:** Demographic profile of respondents.

	n	%		n	%
Gender			Age Group		
Male	308	45.0	18–22 years	159	23.2
Female	376	55.0	23–26 years	224	32.7
Total	684	100.0	27–30 years	217	31.7
			Above 30 years	84	12.3
Education			Total	684	100.0
Undergraduate student	576	84.2			
Graduate student	87	12.7	University		
Doctoral student	21	3.1	Fuzhou Institute of Technology	154	22.5
Total	684	100	Guangxi Medical University	90	13.2
			Hunan University	154	22.5
Income			South China Normal University	144	21.1
Below CNY 2500	17	2.5	South China Agricultural University	142	20.8
CNY 2501–5000	54	7.9	Total	684	100
CNY 5001–7500	94	13.7			
CNY 7501–10,000	283	41.4	Subject		
CNY 10,001–12,500	167	24.4	Social science	482	70.5
Above CNY 12,500	69	10.1	Natural science	202	29.5
Total	684	100	Total	684	100

1 United States Dollar equals 7.20 Chinese Yuan.

### Validity and reliability

Referring to Table 3, PVS recorded the highest mean value. On the other hand, SUN recorded the lowest mean value and the highest standard deviation, which indicated the substantial variation of subjective norms among the respondents. Adding to that, the internal consistency of the instrument was determined based on the values of Cronbach’s alpha coefficient, composite reliability, and Dijkstra-Henseler’s rho. The obtained values exceeded 0.7, which confirmed the reliability of the instrument. The values of average variation extraction (AVE) and factor loadings were also determined. As shown in Table 3, the obtained values of AVE exceeded 0.5, suggesting good convergent validity.

**Table 3:** Reliability and validity.

Variables	Items	Mean	SD	Cronbach’s alpha	Dijkstra-Hensele’s *rho*	Composite reliability	Average variance extracted	Variance inflation factor
RTP	4	3.585	0.916	0.871	0.877	0.913	0.724	1.309
SEF	5	3.683	0.869	0.888	0.893	0.919	0.694	1.334
NFA	5	3.736	0.852	0.883	0.896	0.915	0.684	1.388
PVS	5	3.779	0.853	0.894	0.907	0.923	0.706	1.193
ORG	4	3.696	0.889	0.874	0.903	0.914	0.728	1.378
ATE	5	3.612	0.903	0.893	0.907	0.922	0.704	1.588
SUN	4	3.491	1.033	0.916	0.924	0.941	0.800	1.651
PBC	5	3.541	0.968	0.911	0.921	0.934	0.740	1.659
SEI	5	3.601	0.923	0.901	0.918	0.927	0.718	-

*RTP* risk-taking propensity, *SEF* self-efficacy, *NFA* need for achievement, *PVS* perceived values on sustainability, *ORG* opportunity recognition competency, *ATE* attitude towards entrepreneurship, *SUN* subjective norms, *PBC* perceived behavioural control, *SEI* social entrepreneurial intention.

Fornell–Larcker criterion, heterotrait-monotrait (HTMT) ratio, and cross-loadings were used in this study to measure the discriminant validity of the reflective measurement model items. Based on the results of Fornell-Larcker criterion in Table 4, the diagonal exceeded the recorded correlation coefficients in the corresponding columns, suggesting good discriminant validity. Besides that, recorded values for heterotrait-monotrait (HTMT) ratio were below 0.9, which established good discriminant validity. Referring to Table 5, the external loadings of items on the corresponding constructs exceeded the cross-loadings of items on the other constructs^136^, which confirmed good discriminant validity of the items.

**Table 4:** Discriminant validity.

	RTP	SEF	NFA	PVS	ORG	ATE	SUN	PBC	SEI
Fornell–Larcker criterion									
RTP	0.851								
SEF	0.393	0.833							
NFA	0.432	0.450	0.827						
PVS	0.271	0.311	0.383	0.840					
ORG	0.389	0.388	0.385	0.363	0.853				
ATE	0.461	0.434	0.412	0.300	0.408	0.839			
SUN	0.449	0.389	0.379	0.215	0.374	0.506	0.895		
PBC	0.428	0.411	0.352	0.243	0.384	0.503	0.563	0.860	
SEI	0.510	0.439	0.451	0.348	0.427	0.480	0.509	0.458	0.847
Heterotrait–Monotrait ratio									
RTP	–								
SEF	0.446	–							
NFA	0.490	0.507	–						
PVS	0.305	0.346	0.432	–					
ORG	0.433	0.430	0.432	0.411	–				
ATE	0.518	0.487	0.460	0.337	0.453	–			
SUN	0.502	0.430	0.413	0.232	0.403	0.549	–		
PBC	0.480	0.455	0.385	0.265	0.416	0.550	0.616	–	
SEI	0.571	0.488	0.503	0.389	0.471	0.526	0.551	0.495	–

*RTP* risk-taking propensity, *SEF* self-efficacy, *NFA* need for achievement, *PVS* perceived values on sustainability, *ORG* opportunity recognition competency, *ATE* attitude towards entrepreneurship, *SUN* subjective norms, *PBC* perceived behavioural control, *SEI* social entrepreneurial intention.

**Table 5:** Loading and cross loadings.

Items	RTP	SEF	NFA	PVS	ORG	ATE	SUN	PBC	SEI
RTP1	0.935	0.386	0.433	0.308	0.395	0.416	0.369	0.345	0.471
RTP2	0.822	0.305	0.331	0.192	0.296	0.379	0.386	0.384	0.395
RTP3	0.843	0.302	0.341	0.178	0.317	0.414	0.412	0.389	0.447
RTP4	0.797	0.344	0.364	0.242	0.312	0.358	0.361	0.338	0.420
SEF1	0.385	0.954	0.436	0.333	0.383	0.399	0.324	0.357	0.424
SEF2	0.285	0.788	0.324	0.187	0.261	0.343	0.336	0.360	0.368
SEF3	0.324	0.796	0.365	0.225	0.321	0.356	0.319	0.317	0.331
SEF4	0.342	0.825	0.357	0.254	0.328	0.366	0.367	0.402	0.356
SEF5	0.293	0.791	0.386	0.288	0.317	0.340	0.272	0.275	0.347
NFA1	0.441	0.429	0.953	0.354	0.370	0.403	0.406	0.370	0.442
NFA2	0.376	0.401	0.804	0.283	0.335	0.337	0.369	0.352	0.375
NFA3	0.328	0.379	0.808	0.271	0.318	0.350	0.298	0.261	0.378
NFA4	0.323	0.293	0.788	0.351	0.268	0.326	0.259	0.248	0.337
NFA5	0.303	0.350	0.768	0.331	0.296	0.272	0.206	0.200	0.317
PVS1	0.296	0.330	0.396	0.964	0.355	0.327	0.252	0.267	0.350
PVS2	0.188	0.231	0.262	0.801	0.265	0.218	0.155	0.188	0.284
PVS3	0.194	0.228	0.293	0.796	0.275	0.209	0.145	0.164	0.281
PVS4	0.226	0.238	0.291	0.805	0.314	0.232	0.169	0.198	0.262
PVS5	0.222	0.268	0.354	0.823	0.310	0.264	0.170	0.194	0.275
ORG1	0.431	0.423	0.385	0.319	0.944	0.453	0.436	0.449	0.445
ORG2	0.316	0.289	0.273	0.287	0.813	0.327	0.291	0.321	0.341
ORG3	0.320	0.327	0.365	0.348	0.845	0.303	0.287	0.277	0.364
ORG4	0.223	0.255	0.274	0.283	0.802	0.279	0.221	0.221	0.279
ATE1	0.479	0.425	0.390	0.248	0.385	0.956	0.548	0.535	0.475
ATE2	0.407	0.354	0.352	0.254	0.348	0.823	0.469	0.449	0.450
ATE3	0.367	0.343	0.349	0.246	0.323	0.841	0.399	0.405	0.373
ATE4	0.335	0.354	0.335	0.226	0.320	0.793	0.380	0.374	0.347
ATE5	0.325	0.337	0.296	0.297	0.331	0.769	0.288	0.317	0.350
SUN1	0.445	0.406	0.397	0.258	0.381	0.506	0.957	0.539	0.513
SUN2	0.416	0.326	0.323	0.151	0.343	0.435	0.865	0.497	0.435
SUN3	0.353	0.329	0.321	0.162	0.312	0.421	0.872	0.500	0.422
SUN4	0.388	0.323	0.309	0.190	0.296	0.442	0.882	0.477	0.447
PBC1	0.425	0.428	0.360	0.260	0.387	0.498	0.538	0.969	0.461
PBC2	0.330	0.339	0.308	0.244	0.320	0.422	0.410	0.799	0.362
PBC3	0.375	0.373	0.313	0.215	0.324	0.413	0.508	0.849	0.409
PBC4	0.336	0.300	0.239	0.123	0.295	0.404	0.473	0.836	0.362
PBC5	0.365	0.313	0.283	0.193	0.317	0.420	0.485	0.838	0.364
SEI1	0.526	0.448	0.427	0.297	0.417	0.513	0.573	0.531	0.949
SEI2	0.401	0.348	0.320	0.276	0.329	0.359	0.413	0.351	0.832
SEI3	0.390	0.362	0.391	0.263	0.348	0.357	0.400	0.316	0.810
SEI4	0.400	0.356	0.408	0.324	0.372	0.402	0.371	0.339	0.818
SEI5	0.425	0.333	0.359	0.320	0.334	0.377	0.367	0.364	0.820

*RTP* risk-taking propensity, *SEF* self-efficacy, *NFA* need for achievement, *PVS* perceived values on sustainability, *ORG* opportunity recognition competency, *ATE* attitude towards entrepreneurship, *SUN* subjective norms, *PBC* perceived behavioural control, *SEI* social entrepreneurial intention.

### Hypotheses testing

**Figure 2:**
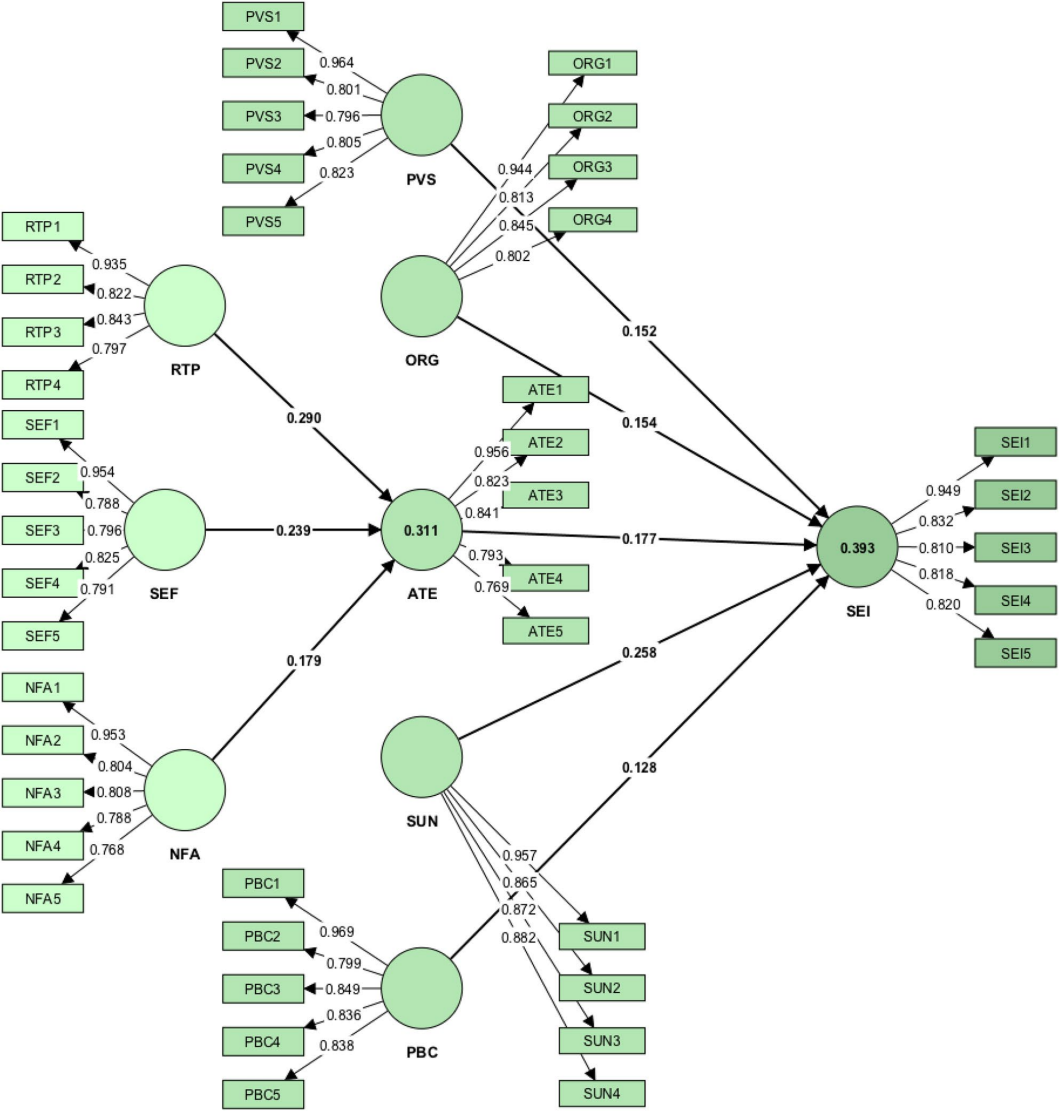
Research framework with findings.

Figure 2 and Table 6 present the overall results of path analysis. Firstly, the results revealed the significant and positive influence of RTP (*β*-value = 0.290, *p*-value < 0.001), SEF (*β*-value = 0.239, *p*-value < 0.001), and NFA (*β*-value = 0.179, *p*-value < 0.001) on ATE. Thus, H1, H2, and H3 were supported. Among these three factors, RTP exhibited the strongest influence on ATE. The exclusion of RTP reduced *R*^2^ by 9.3% (*f*^2^ = 0.093). RTP explained 31.1% of the total variance in ATE (*R*^2^ = 0.311).

**Table 6:** Hypothesis testing.

Hypothesis		Beta	CI (min)	CI (max)	t	*p*	*r*^2^	*f*^2^	*Q*^2^	Decision
Factors affecting attitude towards entrepreneurship										
H_1_	RTP → ATE	0.290	0.230	0.348	8.012	0.000		0.093		Supported
H_2_	SEF → ATE	0.239	0.177	0.303	6.237	0.000	0.311	0.062	N	Supported
H_3_	NFA → ATE	0.179	0.115	0.240	4.683	0.000		0.034		Supported
Factors affecting social entrepreneurial intention										
H_4_	PVS → SEI	0.152	0.098	0.207	4.596	0.000		0.032		Supported
H_5_	ORG → SEI	0.154	0.096	0.215	4.248	0.000		0.028		Supported
H_6_	ATE → SEI	0.177	0.108	0.243	4.326	0.000	0.393	0.033	N	Supported
H_7_	SUN → SEI	0.258	0.192	0.321	6.514	0.000		0.066		Supported
H_8_	PBC → SEI	0.128	0.062	0.197	3.087	0.001		0.016		Supported
Mediating effect of attitude towards entrepreneurship										
H_9a_	RTP → ATE → SEI	0.051	0.028	0.077	3.523	0.000				Supported
H_9b_	SEF → ATE → SEI	0.042	0.023	0.064	3.423	0.000				Supported
H_9c_	NFA → ATE → SEI	0.032	0.016	0.049	3.109	0.001				Supported

*RTP* risk-taking propensity, *SEF* self-efficacy, *NFA* need for achievement, *PVS* perceived values on sustainability, *ORG* opportunity recognition competency, *ATE* attitude towards entrepreneurship, *SUN* subjective norms, *PBC* perceived behavioural control, *SEI* social entrepreneurial intention.

Secondly, the obtained results demonstrated the significant and positive influence of PVS (*β*-value = 0.152, *p*-value < 0.001), ORG (*β*-value = 0.154, *p*-value < 0.001), ATE (*β*-value = 0.177, *p*-value < 0.001), SUN (*β*-value = 0.258, *p*-value < 0.001), and PBC (*β*-value = 0.128, *p*-value < 0.01) on SEI. Thus, H4, H5, H6, H7, and H8 were supported. Overall, these five factors explained 39.3% of the total variation in SEI (*R*^2^ = 0.393). Besides that, the results proved the significance of SUN as the strongest predictor of SEI. In particular, a one-unit change in SUN would increase SEI by 0.258 units. The exclusion of SUN reduced *R*^2^ by 6.6% (*f*^2^ = 0.066).

Thirdly, the obtained results revealed the direct influence of RTP (*β*-value = 0.051, *p*-value < 0.001), SEF (*β*-value = 0.042, *p*-value < 0.001), and NFA (*β*-value = 0.032, *p*-value < 0.01) on SEI. Referring to the recommendation by Hair et al.^137^ on the testing of mediating effects, the current study considered the variance accounted for (VAF) values through path coefficients. The results revealed the partial mediating effects of ATE on the relationships of RTP (VAF = 0.502), SEF (VAF = 0.502), and NFA (VAF = 0.498) with SEI. With that, H9a, H9b, and H9c were supported.

Adding to that, the current study examined the predictive relevance of the model based on the coefficient of determination (*R*^2^) and effect size (*f*^2^). Based on the general rule of thumb for *R*^2^ by Hair et al.^138^, the predictive power of ATE (*R*^2^ = 0.311) and SEI (*R*^2^ = 0.393) was between medium and weak. Meanwhile, the effect sizes for most of the constructs were between small (0.02) and medium (0.15), except for PBC (*f*^2^ < 0.02). Besides that, the results confirmed SUN as the strongest endogenous predictor of SEI. Overall, the model demonstrated good predictive relevance.

### Multi-group analysis

This study assessed the measurement invariance of composite models (MICOM) for the compositional invariance assessment. In the permutation test, the recorded *p*-values of all constructs for the case of university subject (i.e., social science versus natural science) did not exceed 0.05, suggesting the absence of compositional invariance. However, all *p*-values of constructs for the case of gender in this permutation test exceeded 0.05, implying the presence of compositional invariance. With that, this study proceeded to compare the standardised path coefficients by gender through MGA in PLS. Referring to the results in Table 7, gender did not lead to the outcome of significant differences for most of the pathways (*p*-value > 0.05). Interestingly, a significant difference between male and female respondents was observed in the relationship between NFA and ATE (*p*-value < 0.01), which suggested that the formation of SEI in men is more significantly affected by NFA than women.

**Table 7:** Multi-group analysis.

Associations		Male (N = 308)		Female (N = 376)		Difference		Decision
		Beta	*p-value*	Beta	*p-value*	Beta	*p-value*	
H_1_	RTP → ATE	0.285	0.000	0.288	0.000	− 0.003	0.484	No difference
H_2_	SEF → ATE	0.209	0.000	0.283	0.000	− 0.074	0.168	No difference
H_3_	NFA → ATE	0.295	0.000	0.076	0.084	0.219	0.002	Difference
H_4_	PVS → SEI	0.185	0.000	0.123	0.004	0.062	0.176	No difference
H_5_	ORG → SEI	0.137	0.003	0.180	0.000	− 0.043	0.277	No difference
H_6_	ATE → SEI	0.140	0.009	0.207	0.000	− 0.068	0.206	No difference
H_7_	SUN → SEI	0.240	0.000	0.266	0.000	− 0.026	0.371	No difference
H_8_	PBC → SEI	0.172	0.001	0.083	0.078	0.089	0.135	No difference

*RTP* risk-taking propensity, *SEF* self-efficacy, *NFA* need for achievement, *PVS* perceived values on sustainability, *ORG* opportunity recognition competency, *ATE* attitude towards entrepreneurship, *SUN* subjective norms, *PBC* perceived behavioural control, *SEI* social entrepreneurial intention.

## Discussion

The significance of social entrepreneurship has gained growing research interest as a solution to the emerging social and environmental issues^139^. With respect to TPB, the current study empirically examined the influence of attitude towards entrepreneurship, subjective norms, and perceived behavioural control on social entrepreneurial intention, as well as the mediating effects of attitude towards entrepreneurship on the relationships of risk-taking propensity, self-efficacy, and need for achievement with social entrepreneurial intention. This study intended to extend TPB with two additional factors, namely perceived values on sustainability and opportunity recognition competency. Chinese university students and graduates were targeted in this study.

This study obtained adequate empirical evidence on the significant and positive influence of risk-taking propensity, self-efficacy, and need for achievement on attitude towards entrepreneurship. Firstly, the obtained results in this study confirmed the significance of risk-taking propensity of Chinese students and graduates as a determinant of their attitude towards entrepreneurship. Previous studies have come to inconsistent conclusions about the relationship between RTP and ATE. For example, Ahmad and Malik^140^ proposed that individual RTP significantly influences attitudes towards entrepreneurship intention. Shukla and Kumar^141^ validated the mediating role of ATE in the relationship between RTP and entrepreneurial intention, while also emphasizing the positive impact of RTP on ATE. However, Mahmood et al.^78^ did not test for a significant effect of RTP on ATE in a sample of Malaysian millennials. Indeed, culture has an influence on the relationship between risk propensity and behaviour. Under the influence of Chinese culture, university students choose more difficult challenges when their risk-taking decisions increase^142^. Therefore, when the propensity for risk-taking increases, Chinese university students' attitudes toward entrepreneurship also increase. Secondly, self-efficacy has been shown to positively influence entrepreneurial attitudes, consistent with the finding of Bouarir et al.^143^ and Sekerbayeva et al.^144^. SEF has the ability to shape the entrepreneurial mindset of university students^144^. In social entrepreneurship, students with higher self-efficacy show more enthusiasm^68^. The study demonstrated that individuals who believe in their own abilities are more likely to have positive attitudes toward entrepreneurship. Thirdly, the study verified that need for achievement is a positive predictor of attitude towards entrepreneurship. Our study is consistent with the results obtained by Dzomonda and Neneh^145^ as well as Bağış et al.^146^. The NFA influences individuals' perceptions of their entrepreneurial careers^145^. It motivates students to aspire to improve their abilities to pursue their goals^77^. The higher the NFA, the more students want to set challenging goals for themselves and solve difficult problems^79^. The results show that students with this characteristic have a more positive attitude towards entrepreneurship. Furthermore, based on the results of mean and standard deviation, the participating Chinese students and graduates in this study demonstrated medium levels of risk-taking propensity, self-efficacy, and need for achievement. In other words, these students and graduates are not afraid of or proactive in taking risks and have neither positive nor negative attitude towards their ability to start a business and the need to gain a sense of achievement from it.

This study also obtained adequate empirical evidence on the significant and positive influence of perceived values on sustainability, opportunity recognition competency, attitude towards entrepreneurship, subjective norms, and perceived behavioural control on social entrepreneurial intention. Firstly, individuals who are high perceived values on sustainability, meaning concerned about sustainable production and consumption, as well as engage in sustainable practices tend to be more concerned about the environmental implications of their behaviour^147^. They demonstrate higher propensity to make decisions that benefit or do not negatively influence the environment^148^. Consequently, they exhibit high level of social entrepreneurial intention. Secondly, we confirmed that the finding of Hoong et al.^94^ about the positive relationship between ORG and SEI are correct. The ability to identify opportunities is crucial because the first step in trying to solve social problems is to identify social needs^31^. Research has demonstrated that individuals who are good at identifying potential opportunities and are keen to learn more about a product or service have higher social entrepreneurial intentions. Limited opportunity recognition abilities hinder individuals' engagement in social entrepreneurship^93^. Moreover, PVS and ORG are proven influencing factors for SEI, suggesting that individuals who form social entrepreneurial intentions will first possess pro-environmental motivation and the ability to contribute to the occurrence of social entrepreneurship.

Prior studies that explored students’ entrepreneurial intention typically explored the significance of opportunity recognition competency, attitude towards entrepreneurship, subjective norms, and perceived behavioural control as antecedent variables^91,97,104,110^. These constructs clearly demonstrated their significance influence of enhancing social entrepreneurial intention among the participating university students and graduates in this study. Firstly, our study, along with those of Yamini et al.^19^ and Handayani et al.^149^, confirmed the positive effect of ATE on SEI. Entrepreneurial attitude is an assessment of social entrepreneurial behaviour^98^. The positive attitude towards social entrepreneurship plays a crucial role in the formation of interest in becoming a social entrepreneur^149^. The finding is not only for general entrepreneurship, but our study also proves that it can be applied in the context of social entrepreneurship. Secondly, the findings show that SUN is a positive influencing factor for SEI. While the same result was obtained in the study of Tiwari et al.^105^as well as Durac and Moga^150^, Handayani et al.^149^ did not find any effect of SUN on SEI. Potential social entrepreneurs in China, living in a culture with a strong collectivist concept, readily succumb to the influence of those around them to develop SEI^103^. The study, along with Tiwari et al.^105^, coincidentally finds that SUN was the strongest of the five influences on SEI, which demonstrates the importance of the role of norms in Chinese culture. The results also suggest that pressure and encouragement from society, family, and friends regarding entrepreneurship can make students think about the possibility of starting a social enterprise. Thirdly, the study also verified the positive effect of PBC on SEI. This finding is consistent with the results reported by Durac nd Moga^150^ as well as Handayani et al.^149^. PBC has been considered the strongest predictor of entrepreneurial intentions and SEI in past studies^105^. Liñán and Chen^96^ even directly defined PBC as the ease of becoming an entrepreneur. This point shows a strong correlation between students’ PBC and their becoming social entrepreneurs, which is also identical to the results of our study. The findings suggest that individuals with strong SEI will perceive themselves as capable of managing a good business, having resources available and having the opportunity to start a business. Basically, the level of SEI is higher when an individual has the goal and confidence of becoming an entrepreneur, receives support from the significant others to pursue social entrepreneurship, and has access to the required resources for social entrepreneurship. The results imply that the TPB is also applicable in the study of SEI, and again demonstrate that TPB can be widely used in the construction of intentional models.

Besides that, the current study empirically demonstrated the significant and positive influence of risk-taking propensity, self-efficacy, and need for achievement on social entrepreneurial intention and the partial mediating effects of attitude towards entrepreneurship on the above relationships. Of these three mediating relationships, only the mediating role of ATE between SEF and SEI has been directly demonstrated^105^. Our study reaffirms this relationship and points to a partially mediating role of ATE. Individuals with stronger SEFs are prone to have positive effect on ATEs, which in turn generate stronger SEI. SEFs can also directly influence SEI because the stronger the SEF, the more confident individuals are in finding social needs and the more willing they are to commercialize them^119^. However, there are no existing studies that directly describe the mediating role of ATE in the relationship between RTP, NFA, and SEI. Our study fills this gap and confirms partial mediating roles of ATE in them. First, the more motivated students were to take risks, the less they feared entrepreneurship and the more pronounced their positive attitudes and tendencies toward social entrepreneurship^61^. Second, NFA as an aspiration that wants to be transformed into the idea of creating a social enterprise requires the help of a good entrepreneurial attitude^125,126^. Based on the obtained results, therefore, students and graduates with high risk-taking propensity, self-efficacy, and need for achievement will possess social entrepreneurial intention, and will form positive attitude towards entrepreneurship prior to the formation of the intention to engage in social entrepreneurship.

Last but not least, the current study found significant difference between men and women in their need to gain a sense of fulfilment from entrepreneurship. Gender differences in entrepreneurship have been a hot topic in the field of entrepreneurship^151^. Vodă and Florea^152^ have found gender differences in the effect of need for achievement on entrepreneurial intentions, with men in particular being more likely to entertain the idea of entrepreneurship due to the pursuit of achievement. However, some studies have also concluded that female university students have greater achievement motivation and achievement needs than male students^153,154^. Our results demonstrate differences between males and females in the relationship between NFA and ATE, and males show a greater effect; however, this difference does not exist in the effect of ATE on SEI. The results suggest that men display higher interest to achieve fame, fortune, and social status than women, which can be achieved through entrepreneurship. The pursue for social status often leads men to be more enthusiastic about entrepreneurship. However, there is no longer a difference between men and women when it comes to transforming entrepreneurial attitudes into social entrepreneurial intentions. This implies that gender plays a minor role in the formation of more specific and strong social entrepreneurial intentions. The bias in our results compared to previous studies may be due to the fact that the study focused on university students and graduates in southern China, where men are more concerned about being successful in their careers due to traditional beliefs, and therefore the influence of the need for achievement is more pronounced in the male population. Surprisingly, this study did not find any differences in the other influential factors among the participating students and graduates with different university subjects (i.e., social science versus natural science).

## Implications

### Theoretical implications

This study contributes to the existing literature on social entrepreneurship in some ways. The first and most important contribution is the inclusion of the study of perceived values on sustainability and opportunity recognition competency in the field of social entrepreneurial intentions. The present study attempts to empirically demonstrate how perceived values on sustainability and opportunity recognition competency encourage individuals to generate ideas for social entrepreneurship through our proposed model. We hope that the results of this study will reveal how pro-environmental values and abilities that are beneficial to entrepreneurship contribute to understanding the formation of social entrepreneurial intentions.

Based on the basic framework of the Theory of Planned Behavior, this study further expands the antecedents of social entrepreneurial intention based on the existing literature. First, this study extends the application of TPB in the field of social entrepreneurial intention research. We attempt to identify additional influences at the individual level and construct and validate a modified model to explain the formation of social entrepreneurial intentions, providing new insights for social entrepreneurship research. Second, the study further contributes to the growing literature focusing on the role of individual psychological dispositions of men and women in their entrepreneurial preparation activities.

### Practical implications

In addition to theoretical insights, this study has practical implications for the development and promotion of social entrepreneurial intentions among university students and the general population. First, government and universities should focus on risk-taking propensity, self-efficacy and need for achievement. research has demonstrated the predictive effects of these three factors on attitudes towards entrepreneurship. Policymakers should provide more safeguards against entrepreneurial risk to increase the confidence of potential entrepreneurs in dealing with risk, making them more risk-averse and increasing their risk propensity. Universities can help university students increase their entrepreneurial confidence and boost their self-confidence by conducting some courses and implementing some programs. At the same time, the cultivation of the need for achievement requires not only the development of appropriate strategies by universities, but actually the desire to pursue achievement needs to be conveyed before the university stage and even during the process of personal growth. These will effectively contribute to enhance the entrepreneurial attitude of individuals to become positive.

Second, perceived values on sustainability and opportunity recognition competency are important validations that distinguish this study from other TPB-based studies on social entrepreneurial intentions and may be worthier of attention by potential entrepreneurs and policy makers. Both universities and governments should make efforts in promoting sustainability values. The government needs to develop policies targeted at enhancing sustainability and help individuals develop pro-environmental values. Universities, on the other hand, should do a better job of promoting relevant policies to convey to students the idea of protecting the environment and helping sustainability. In addition to values-based training, universities should provide courses, lectures, and hands-on activities to enhance entrepreneurial skills (especially opportunity recognition) to increase students’ motivation to participate in social entrepreneurship practices. These measures and actions will have a positive impact on the intention of individuals to engage in social entrepreneurship activities.

Third, this study also re-emphasizes the positive and significant effects of attitude towards entrepreneurship, subjective norm and perceived behavioural control on social entrepreneurial intentions University administrators and policy makers must pay attention to entrepreneurial attitudes because it is the most direct factor that makes It is important for university administrators and policy makers to pay attention to entrepreneurial attitudes because it is the most direct factor in generating social entrepreneurial intentions. They should develop strategies and measures to help students and the general population develop the right entrepreneurial attitudes and mindsets. These correct mindsets and attitudes encourage individuals to be innovative and expect results. Universities and governments should also provide a supportive environment for students to develop social entrepreneurship ideas and related knowledge and skills. These can contribute to the enhancement of students' subjective norms. In addition, perceived behavioural control needs to be addressed. This can be done by conducting training sessions and programs to enhance individual confidence in entrepreneurship. Universities can also arrange social entrepreneurship mentors for students to help them have the opportunity to increase their entrepreneurial contacts and resources for their social entrepreneurial activities.

## Conclusions

As a growing research area in entrepreneurship, social entrepreneurship plays a significant role and yields substantial benefits. Firstly, new businesses increase employment opportunities, promote economic development, and develop individual employability and entrepreneurial skills^155^. Secondly, these new businesses effectively deal with social inequalities, promote social efficiency, and promote sustainable use of resources^156^. Considering that, the development of social entrepreneurship in developing countries is deemed essential, especially with the prevalence of social issues like poverty and inequality^29^.

Exploring factors that influence social entrepreneurial intention provides valuable insights on why individuals decide to pursue social entrepreneurship. Addressing that, the current study demonstrated the significance of perceived values on sustainability, opportunity recognition competency, attitude towards entrepreneurship (as predicted by risk-taking propensity, self-efficacy, and need for achievement), subjective norms, and perceived behavioural control in developing social entrepreneurial intention. The findings are aligned with the TPB framework. Moreover, this study empirically proved the partial mediating effects of attitude towards entrepreneurship on the relationships of taking propensity, self-efficacy, and need for achievement with social entrepreneurial intention. Furthermore, this study found that gender can lead to the differences in the formation of NFA-ATE pathways—men are more likely to have favourable attitude towards entrepreneurship than women due to their pursuit of achievement.

The current study encountered several limitations. Firstly, this study conveniently sampled 684 university students and graduates from five selected universities in Southern China only for an online survey. The convenient sampling strategy is generally easier to implement, but the selected sample may not evenly distribute. The limited sampling area (Southern China) and the inclusion of university graduates in this study resulted in a sample that was not fully representative of the target population (Chinese university students). Therefore, it is recommended for future research to consider a larger random sample across different regions in order to obtain a more representative sample of Chinese university students. Secondly, this study expected that university subjects contribute significant differences in the influence of certain determinants of social entrepreneurial intention. However, the obtained results proved otherwise. The courses taken or the values developed by students in different subjects may not exhibit any significant influence on their social entrepreneurial intention. Nonetheless, this study noted the possibility that the sample selection or insufficient subject differentiation may contribute to different outcomes. Therefore, it is recommended for future research to have a more explicit comparison on whether there are differences in social entrepreneurial intention among students from different disciplines (e.g., business and engineering). Thirdly, the research methods we used have limitations. the PLS-SEM may not be sufficient to face the complexity of social sciences. Future scholars can use more appropriate data methods to validate our model. Finally, this study suggests that the difference between males and females in the effect of achievement needs on entrepreneurial attitudes stems from the influence of Chinese culture, but this has not been confirmed. Future research is expected to focus on whether culture can explain the gender-induced differences.

### Supplementary Information

Supplementary Table S1.

Supplementary Information.

## Data Availability

The original contributions presented in the study are included in the article/Supplementary Material 2. Dataset, further inquiries can be directed to the corresponding author/s.
