# Supplementary material for: Social entrepreneurial intention among university students in China
Source: Sci Rep. 2024 Mar 28;14:7362. doi: 10.1038/s41598-024-58060-4 (PMC10978997; doi:10.1038/s41598-024-58060-4)
Supplement: Supplementary file 1 — Supplementary Table S1. [file 41598_2024_58060_MOESM1_ESM.docx]

**Table S1.** Survey Instrument

| Code | Questions |
| --- | --- |
| RTP1 | When it comes to picking a work environment, I am ready to take any risk. |
| RTP2 | I prefer a high-risk, high-reward work environment. |
| RTP3 | Even if the current workplace offers excellent benefits, I would prefer the opportunity to work in a new setting with unknown challenges. |
| RTP4 | Job-related challenges are something I never tried to avoid. |
| SEF1 | If I work hard enough, I can always tackle challenging difficulties. |
| SEF2 | It is simple for me to stick to my objectives and achieve my objectives. |
| SEF3 | I am confident in my ability to deal well with unforeseen circumstances. |
| SEF4 | I know how to deal with unforeseen events thanks to my resourcefulness. |
| SEF5 | If I put forth the necessary effort, I can solve most challenges. |
| NFA1 | I am pleased when I can take on added job responsibilities. |
| NFA2 | I like to set challenging goals for myself on the job. |
| NFA3 | I enjoy situations at work where I am personally responsible for finding solutions to problems. |
| NFA4 | I try very hard to improve on my past performance at work. |
| NFA5 | I get the most satisfaction when completing job assignments that are fairly difficult. |
| PVS1 | I feel that it is critical to live in harmony with other animals and nature by implementing sustainable production and consumption practices. |
| PVS2 | Sustainable production and consumption, in my opinion, is the best way to safeguard natural resources. |
| PVS3 | I feel that household sustainability measures (e.g., minimizing food, water, electricity, natural gas, and throwaway products waste) are critical to the planet's survival. |
| PVS4 | Sustainable restaurant practices (e.g., lowering food, water, power, natural gas, and disposable products waste) are critical, in my opinion, to saving the earth. |
| PVS5 | Environmentally responsible consumption, on the whole, provides good value for money. |
| ORG1 | While going about routine day-to-day activities, I see potential new venture ideas all around me. |
| ORG2 | “Seeing” potential new venture opportunities does not come very naturally to me. |
| ORG3 | I can’t succeed in entrepreneurship if I don’t have a good knowledge of the product or service of any firm. |
| ORG4 | Recognizing good opportunity usually requires experience in a specific industry or marketplace. |
| ATE1 | My professional goal is to be an entrepreneur. |
| ATE2 | Being an entrepreneur is attractive to me. |
| ATE3 | Being an entrepreneur would give me great satisfaction. |
| ATE4 | Being an entrepreneur implies more advantage than disadvantageous to me. |
| ATE5 | If I had the opportunity and resources, I would love to start a business. |
| SUN1 | I'm under a lot of social pressure to start my own business. |
| SUN2 | Most of my close friends and family members want me to start my own business. |
| SUN3 | The majority of my family members want me to start my own business. |
| SUN4 | The majority of my close acquaintances believe I should go into business for myself. |
| PBC1 | I am confident that I can start my own business. |
| PBC2 | I see myself as capable of managing my own enterprise. |
| PBC3 | I have the resources to start my own business. |
| PBC4 | I have all the support I need to run my own business. |
| PBC5 | There are likely to be plenty of opportunities for me to start my own business. |
| SEI1 | I intend to start a social enterprise to promote social equalities. |
| SEI2 | I will always try to make efforts to curtail the social inequalities in the future. |
| SEI3 | I plan to use my knowledge and skills to uplift the community welfare. |
| SEI4 | I would be willing to develop a social organization building prosper society. |
| SEI5 | I can predict that I will engage myself in bridging the social differences in my community. |

Note: RTP: Risk-Taking Propensity; SEF: Self-Efficacy; PAP: Proactive Personality; NFA: Need for Achievement; PVS: Perceived Values on Sustainability; ORG: Opportunity Recognition Competency; ATE: Attitude towards Entrepreneurship; SUN: Subjective Norms; PBC: Perceived Behavioural Control; SEI: Social Entrepreneurial Intention.
